# Curcumin and cinnamon mitigates lead acetate-induced oxidative damage in the spleen of rats

**DOI:** 10.3389/fphar.2022.1072760

**Published:** 2023-01-16

**Authors:** Mahmoud Abdelghaffar Emam, Sameh Mohamed Farouk, Ahmed Aljazzar, Abeer A. Abdelhameed, Abeer A. Eldeeb, Fatma Abdel-monem Gad

**Affiliations:** ^1^ Histology Department, Faculty of Veterinary Medicine, Benha University, Benha, Egypt; ^2^ Cytology and Histology Department, Faculty of Veterinary Medicine, Suez Canal University, Ismailia, Egypt; ^3^ Pathology Department, Collage of Veterinary Medicine, King Faisal University, Al-Hofuf, Saudi Arabia; ^4^ Clinical Pharmacology Department, Faculty of Medicine, Benha University, Benha, Egypt; ^5^ Clinical Pathology Department, Faculty of Veterinary Medicine, Benha University, Benha, Egypt

**Keywords:** lead, cinnamon, curcumin, splenotoxicity, oxidative stress

## Abstract

Lead toxicity is a common occupational and environmental health hazard that exerts many toxic effects on animals and humans, including immunotoxicity. Curcumin (CUR) and cinnamon (CIN) are common medicinal herbs with immunostimulatory and antioxidant properties. Therefore, this study investigated the protective effect of curcumin and cinnamon against lead acetate (LA)-induced splenotoxicity in rats *via* hemato-biochemical, immunological, oxidative stress marker, CYP-2E1 expression, histological, and immunohistological evaluations. Four groups of seven rats each were used: the control group received corn oil as a vehicle; the lead acetate group received (100 mg/kg), the CUR + LA group received curcumin (400 mg/kg) plus lead acetate, and the CIN + LA group received cinnamon (200 mg/kg) plus lead acetate orally for 1 month. LA exposure induced macrocytic hypochromic anemia, leukocytosis, neutrophilia, monocytosis, and lymphopenia. Additionally, significant elevations in serum iron, ferritin levels, and transferrin saturation percentage with significant decline of total and unsaturated iron binding capacities (TIBC and UIBC), transferrin, and immunoglobulin G and M levels were recorded. In addition, lead acetate significantly upregulated splenic CYP-2E1 expression, that was evident by significant depletion of reduced glutathione (GSH) activity and elevation of malondihyde (MDA), nitric oxide (NO), and protein carbonyl (PC) concentrations in the spleen. Histologically, hyperplasia of lymphoid follicles, hemosiderin deposition, and disturbance of CD3 and CD68 immuno-expressions were evident in the spleen from the lead acetate group. However, curcumin and cinnamon administration restored the hemato-biochemical, immunological, and oxidative stress parameters as well as histological and immunohistological pictures toward normalcy. In conclusion, curcumin and cinnamon can partially ameliorate LA-induced oxidative damage in the spleen, possibly through their antioxidant, immunomodulatory, and gene-regulating activities.

## 1 Introduction

The spleen contains about one-fourth of the lymphocytes in the mammalian body, so it is considered the largest secondary immune organ (Cesta, 2006). Histologically, splenic parenchyma is formed of red and white pulps. The former is a blood filter and storage site for iron, erythrocytes, and platelets. However, lymphocytes of the white pulp initiate immune responses to blood-borne antigens (Lewis et al., 2019).

Lead is a common occupational and environmental pollutant that has many toxic effects on animals and humans, including immunotoxicity (Aldahmash and El-Nager, 2016). Lead is a widely used heavy metal in various industrial activities, like the production of paints, batteries, ceramics, motor and electric vehicles, electronic technologies, pipes, tobacco smoke, and printing of books (Mishra et al., 2003).

Contaminated drinking water, food, and air are considered the main sources for lead poisoning (Ferreyra et al., 2009). About 30%–40% of inhaled lead is directly absorbed into the bloodstream, while absorption of ingested lead varies depending on age and nutritional status (Patrick, 2006). About 95% of circulating lead is present in erythrocytes, interfering with their proper functions (Chwalba et al., 2018).

In spite of the exact mechanism by which lead causes immunotoxicity still remaining unknown, some studies have reported its adverse effects.

The target immune cells for lead are T cells (Mishra, 2009) and macrophages (Chen et al., 1997). Exposure to lead impairs the lymphocyte’s function (Dyatlov and Lawrence, 2002) and production of cytokines (Chen et al., 2004) and immunoglobulins (Dietert and Piepenbrink, 2006). Generally, lead induces cellular injury and oxidative stress. It causes an imbalance between free radical generation and the antioxidant defense system (Flora et al., 2007), leading to generation of reactive oxygen species (ROS), including hydrogen peroxide and singlet oxygen, resulting in damage to DNA and membrane lipids (Alhusaini et al., 2019) and damage of mitochondria and lysosomes (Zarei et al., 2017). Lead also interrupts enzyme activation, sulfhydryl protein synthesis, and calcium homeostasis, in addition to decreasing trace mineral absorption and antioxidant reserves in the body (Lynpatrick, 2006).

Recently, significant attention has been paid to the use of herbal products rather than synthetic ones in attenuating the toxic impact of pollutants (Alkhedaide, 2016; Farouk et al., 2021a; 2021b). Among these herbs are curcumin (CUR) and cinnamon (CIN).

CUR is a food spice and coloring agent that has been used in traditional herbal medicines for many centuries (Yadollahi and Zargaran, 2019). Its therapeutic properties are owing to its anti-oxidant, anti-inflammatory, anti-cancer, anti-fibrotic, anti-diabetic, and anti-neurotoxic effects (El-Maddawy and El- Sayed 2018; Emam and El-Shafey 2018; Abdelhamid et al., 2020). CUR reduces oxidative stress *via* scavengers of ROS, reduction of lipid peroxidation, upregulation of antioxidant protein biosynthesis, and inhibition of inflammatory cytokines (Hosseini and Hosseinzadeh, 2018). Additionally, it can chelate oxidative metal ions, including lead, which consequently protects and repairs the cells (Soliman et al., 2015). Although several works of literature have described the protective role of CUR against LA-induced toxicity of the liver (García-Niño and Pedraza-Chaverri, 2014; Soliman et al., 2015; Abdelhamid et al., 2020), kidney (Soliman et al., 2015), and testis (Sudjarwo et al., 2017; Abdelhamid et al., 2020), there is a shortage of studies on the spleen.

On the other hand, CIN is an herbal medicinal plant that is a famous condiment in many foods. Cinnamaldehyde is the main active principle of CIN, which has anti-inflammatory, anti-oxidant, and anti-diabetic effects (Subash-Babu et al., 2014; Lee et al., 2018). In addition, it can be used as an antidote against natural or chemical toxicity (Dorri et al., 2018). CIN is considered a stimulator for antioxidant enzyme activities (Kim et al., 2006), along with acting as an ROS scavenger, metal chelator, and enzyme modulator (Rice-Evans et al., 1997). To our knowledge, the protective effect of CIN against LA-induced testicular toxicity was proven (Elgawish and Abdelrazek, 2014). However, the effect of CIN on LA-induced splenotoxicity has not been studied.

Depending on this background, our study was designed to evaluate the potential protective impact of CUR and CIN against splenic toxicity induced by LA exposure. Hematological, serum iron and immunoglobulin parameter, oxidative stress marker, CYP-2E1 expression, splenic histology, and CD3 and CD68 immunohistochemical expressions were evaluated.

## 2 Materials and methods

### 2.1 Chemicals

LA and CUR were obtained from El-Gomhoria Company, Egypt. CIN barks were purchased from a local market, El-Azhar, Cairo, Egypt. All these materials were freshly prepared before use by dissolving LA and CIN in distilled water (Morgan et al., 2014; Abd Elrasoul et al., 2020), while CUR, a bright yellow powder, was dissolved in corn oil (Sudjarwo et al., 2017) due to its insolubility in water. Diagnostic kits for estimation of malondihyde (MDA), nitric oxide (NO), and reduced glutathione (GSH) were obtained from Biodiagnostic Company (Cairo, Egypt). Protein carbonyl (PC), immunoglobulin G (IgG), and immunoglobulin M (IgM) were purchased from MyBioSource Company (Giza, Egypt). Meanwhile, iron, ferritin, and total iron binding capacity were obtained from Spectrum Company (Germany).

### 2.2 Preparation of CIN aqueous extract

CIN barks were identified by the Faculty of Agriculture, Benha University, Egypt. It was prepared as described by Morgan et al. (2014). The dried CIN was ground into a fine powder and then soaked in distilled water (10 gm/100 ml) for 2 h at 90°C. Next, the solution was filtered, and the filtrate was dehydrated in a hot-air oven at 80°C overnight to obtain a dark reddish brown dry extract. The yield concentration of CIN aqueous solution was about 20% (w/w).

### 2.3 Animals

Twenty-eight mature male Albino rats (weighing 180–200 g) were obtained from the animal house, Faculty of Veterinary Medicine, Benha University, Egypt. The rats were kept under an appropriate environment of temperature (25 ± 2 C), humidity (60%–70%), light (12-h dark/light cycles), and with free access to water and a commercial pellet diet.

### 2.4 Experimental design

Both experimental design and animal handling were ethically approved by the Committee of Research Ethics Board at the Faculty of Veterinary Medicine, Benha University, Egypt (Approval No. BUFVTM 17–03-22). After a 2-week acclimatization period, the rats were randomly divided into four groups (7 rats in each group) in separate cages. The first group served as control animals and received 0.5 ml corn oil, the vehicle of CUR; the second group received LA at a dose of 100 mg/kg (Abd Elrasoul et al., 2020); the third group received LA + CUR where CUR was given at a dose of 400 mg/kg (Sudjarwo et al., 2017) plus LA as in the second group; and the fourth group received LA + CIN where CIN was given at a dose of 200 mg/kg (Morgan et al., 2014) plus LA as in the second group. The experiment was extended for a month, and all animals received their treatments orally once daily using a gavage tube.

#### 2.4.1 Blood collection, serum, and tissue preparations

After 24 h from the last treatment, blood samples were collected from the retro-orbital venus plexus of each animal and divided into two parts. The first part was collected into clean dry tubes containing EDTA for both erythrogram and leukogram including red blood cell count (RBC), hemoglobin concentration (Hb), packed corpuscular volume (PCV), mean corpuscular volume (MCV), mean corpuscular hemoglobin (MCH), mean corpuscular hemoglobin concentration (MCHC), total white blood cell count (WBC), and differential leukocytic count. Meanwhile, the second part of the blood was collected into plain tubes for serum separation, and blood was allowed to coagulate at room temperature for 20 min and then centrifuged at 5,000 rpm for 10 min. The separated sera were carefully collected and stored in deep freeze (−20°C) for biochemical assays.

The rats were killed by cervical decapitation under light anesthesia (ketamine–xylazine mixture, .15 ml/100 g BW/IP). A midline abdominal incision was performed to expose their viscera. The spleen of each rat was excised, cleaned from their surrounding tissues, washed with normal saline, and then prepared for oxidative stress parameter, detection of lead concentration, gene expression of CYP-2E1, and histological and immunohistochemical assessments. Small cross-sections of the spleen were fixed in 10% neutral buffered formalin for histological and immunohistochemical examinations; however, the rest of the spleen was wiped dry with a filter paper and quickly fragmented into small specimens. The portion was homogenized in potassium phosphate buffers (5 ml buffer of PH 7.4/0.5 g tissue), centrifuged at 5,000 rpm for 10 min at 4°C, and then the collected supernatant was stored at −20°C until further assessment of oxidative stress parameters. Another small spleen sample was preserved for gene expression of CYP-2E1 at −80°C. Other spleen specimens were used for detection of lead concentration.

### 2.5 Hematological analyses

The hematological analyses, including erythrogram and leukogram, were determined using an automatic cell counter (CLINDIAG-HA-VET, Belgium) according to Thrall et al. (2012).

### 2.6 Assessment of iron parameters

Serum samples were used for detection of iron parameters, including iron, total iron binding capacity (TIBC) (Fairbanks and Klee, 1987), and ferritin (Blackmore et al., 2008). Other iron parameters, including unsaturated iron binding capacity (UIBC), serum transferrin (Tf), and serum transferrin saturation % (TS%), were calculated as described by Burits and Burns (2014) and Fairbanks and Klee (1987).

### 2.7 Measurement of serum immunoglobulin

Serum samples were used for measurement of IgG and IgM according to the manufacturer’s instructions.

### 2.8 Evaluation of oxidative stress/antioxidant parameters

MDA, NO, PC, and GSH were measured in splenic tissue homogenates according to Ohkawa et al. (1979), Green et al. (1982), Levine et al. (1990), and Beutler et al. (1963), respectively.

### 2.9 Determination of lead content in spleen tissue

Lead concentration in splenic tissues was analyzed using the flame atomic absorption spectrophotometer as the method reported by Szkoda and Zmudzki (2005).

### 2.10 Quantitative analysis of gene expression of CYP-2E1 by real-time PCR (qRT-PCR)

Total cellular RNA was extracted from splenic tissue using an RNeasy Mini kit (Qiagen, Valencia, CA, United States) according to the manufacturer’s instructions. The concentration and purity of RNA were measured by a nanodrop spectrophotometer.

The extracted RNA was reverse-transcribed into complementary DNA (cDNA) using the QuantiTect Reverse Transcription Kit (Applied Biosystem) following the manufacturer’s instructions.

Real-time qPCR amplification and analysis were performed using Applied Biosystem real-time PCR system software to measure the expression of mRNAs of the target gene in the spleen, with B-actin as an internal reference (housekeeping gene). The isolated cDNA was amplified using the SYBR Green Master Mix (*Applied Biosystems*) following the manufacturer’s protocol. The primers used in the amplification are shown in Table 1 based on NBCI rat sequences. The result was calculated using the comparative cycle threshold (Ct) method. All values were normalized to *ß*-actin and reported as fold change over background levels detected in the treated groups. All these steps were performed according to the methods of Livak and Schmittgen (2001).

**TABLE 1:** Primer sequence of the studied rat genes.

Gene	Primer sequence
Cytochrome P450 2E1	Forward: 5′- TTT​GGA​TCC​AAT​GGG​TGA​TGT​TGA​G -3
	Reverse: 5′-TTT​GAA​TTC​CTC​ATT​AGT​AGC​TTT​TTT​GAG-3
β-actin	Forward primer: 5′-GGT​CGG​TGT​GAA​CGG​ATT​TGG -3
	Reverse primer: 5′- ATG​TAG​GCC​ATG​AGG​TCC​ACC-3

### 2.11 Histological evaluation

After fixation in 10% neutral buffered formalin for 48 h, the splenic tissue specimens were dehydrated in increasing concentrations of ethyl alcohol, cleaned using xylene, blocked in paraffin wax, sectioned at 5-μm thicknesses, and stained with hematoxylin and eosin (H&E) according to Bancroft and Gamble (2008).

### 2.12 Immunohistochemical evaluation

For detection of CD3 and CD68, paraffin sections were mounted on positively charged slides and exposed to the antigen retrieval process in citrate buffer (pH 6.0) using a microwave for 10 min. Subsequently, endogenous peroxidase was blocked using H_2_O_2_ for 30 min. The slides were incubated with mouse monoclonal anti-CD3 and anti-CD68 at a dilution of 1:250 (catalog numbers sc-20047 and sc-20060, respectively, Santa Cruz Biotechnology Inc., CA, United States) overnight at 4 °C. After that, the sections were treated with secondary antibodies associated with the streptavidin–biotin–peroxidase complex. Diamino-benzidine (DAB) was used as a chromogen. All tissue sections were counter-stained with hematoxylin.

### 2.13 Histological and immunohistochemical scoring methods

According to Meyerholz and Beck (2019), the ordinal scoring method was used for grading the histological changes in splenic tissues among the different groups. The scores were from 0 to 4, where 0 was normal, 1 was <25% affection, 2 was 25%–50% affection, 3 was 50%–75% affection, and 4 was >75% affection. However, quantitative immunohistochemical analysis of CD3 and CD68 expressions in all the examined splenic tissues was scored using ImageJ 1.47 software (National Institutes of Health, Bethesda, United States) as outlined by Smolen (1990), where the brown color intensities were expressed as the relative optical density of the DAB reaction.

Five random images per slide (*n*=3) at 400X were checked blindly for histological and immunohistochemical scoring using the Leica DM3000 microscope.

### 2.14 Statistical analysis

The statistical analysis was performed using SPSS (SPSS Inc., Chicago, Illinois, USA). All data points were represented as mean values ±SE. Differences between the groups were analyzed using one-way ANOVA (analysis of variance) and *post hoc* Tukey’s tests. *p* values <.05 were regarded as significant.

## 3 Results

### 3.1 Hematological analyses

**FIGURE 1:**
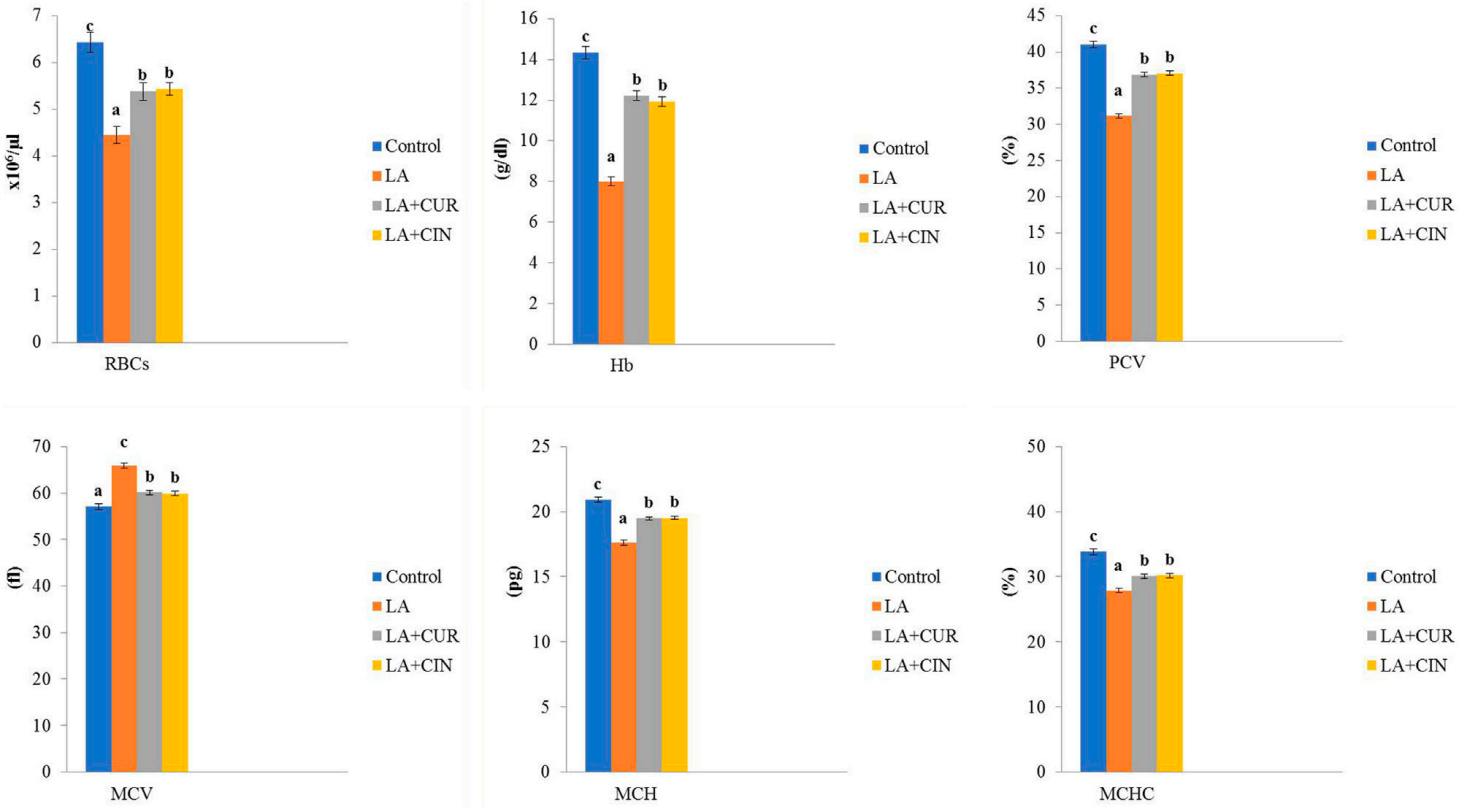
Changes of erythrogram in different treatment groups compared to the control (mean ± SE). Different superscript letters in each figure indicate significant differences at *p* < .05.

In regard to the hemogram, there were significant reductions in RBC count, Hb concentration, and hematocrit percentage in the LA-treated group compared to the control group. Meanwhile, those parameters revealed significant elevations in the LA + CUR and LA + CIN groups compared to the LA-treated group. Concerning red blood cell indices, MCV showed a significant increase with significant decreases in MCH and MCHC in the LA-treated group compared to the control one. However, the LA + CUR and LA + CIN groups exhibited a significant decrease in MCV along with significant increases in MCH and MCHC compared to the LA-treated group (Figure 1).

**FIGURE 2:**
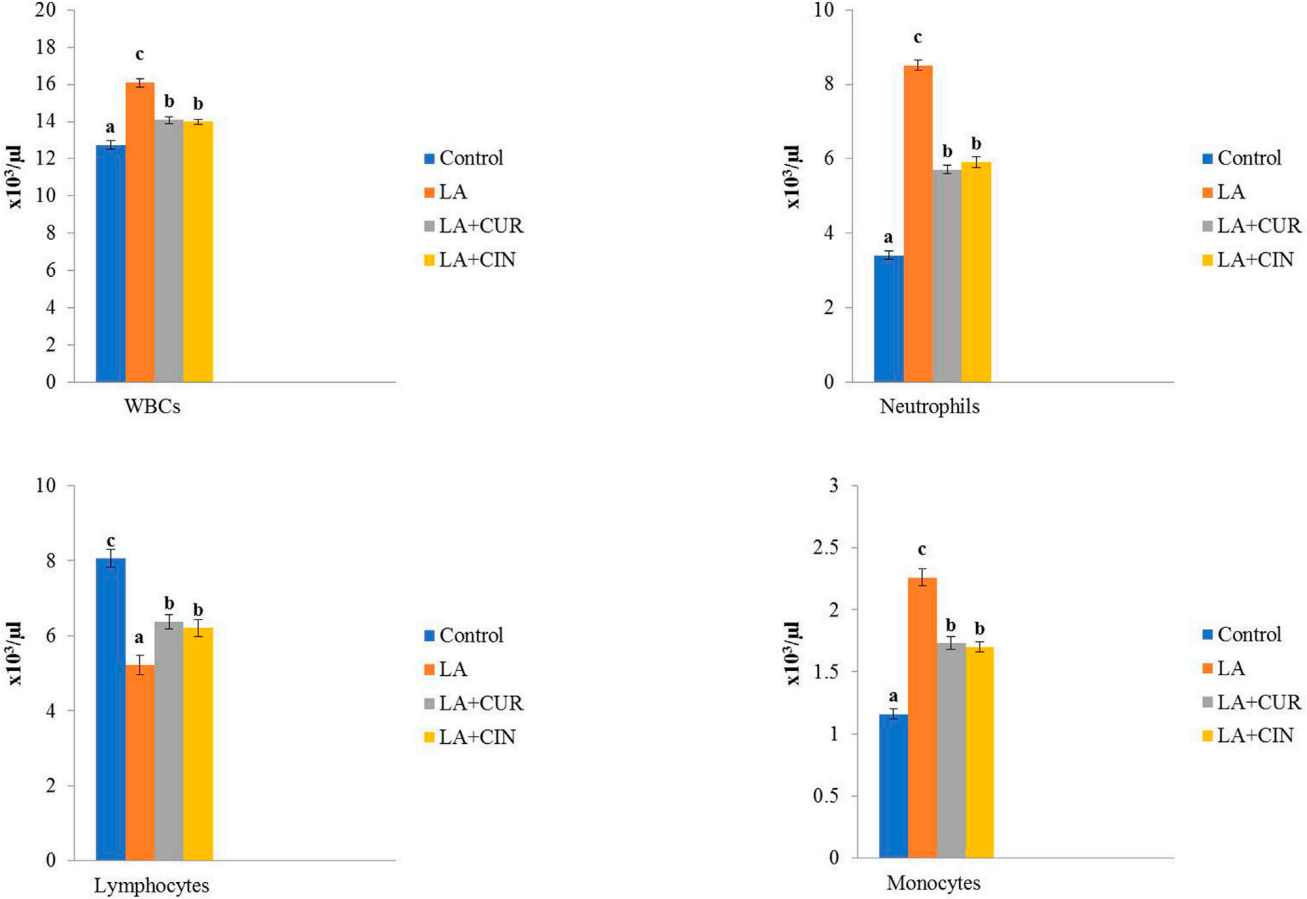
Changes of leukogram in different treatment groups compared to the control (mean ± SE). Different superscript letters in each figure indicate significant differences at *p* < .05.

As shown in Figure 2, leukogram results revealed significant elevations in WBC, neutrophil, and monocyte counts and a significant reduction in lymphocyte count in the LA-treated group compared to the control group. In contrast, the LA + CUR and LA + CIN groups showed significant decreases in WBC, neutrophil, and monocyte counts with a significant increase in lymphocyte count compared to the LA-treated group.

From these results, we concluded that LA induced macrocytic hypochromic anemia with leukocytosis, neutrophilia, monocytosis, and lymphopenia. On the other hand, groups treated by CIN and CUR reversed the bad effect of LA on erythrogram and leukogram.

### 3.2 Iron parameters

**FIGURE 3:**
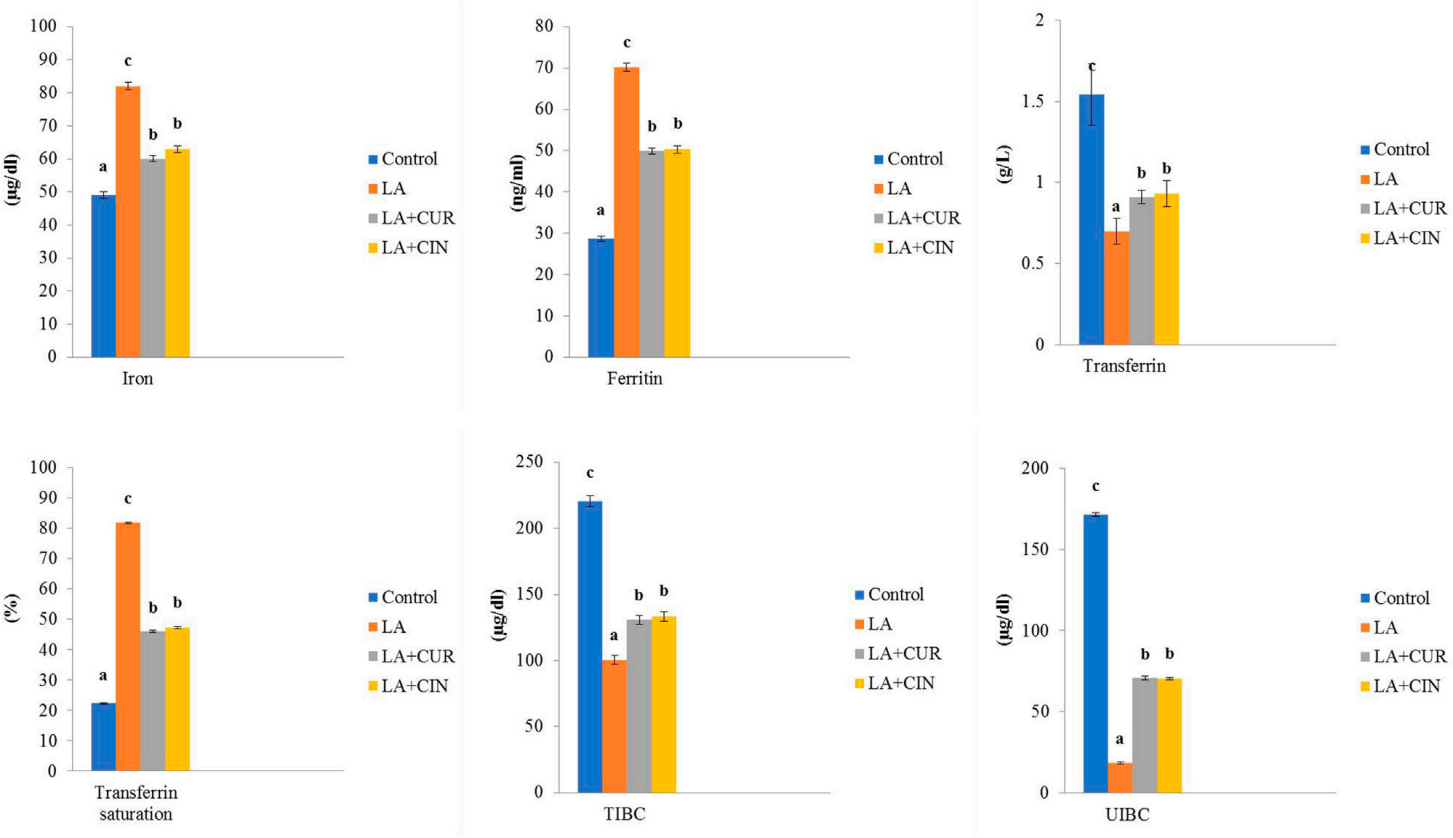
Changes of iron parameters in different treatment groups compared to the control (mean ± SE). Different superscript letters in each figure indicate significant differences at *p* < .05.

Significant elevations in serum iron and ferritin levels, as well as transferrin saturation percentage, along with significant decreases in TIBC, UIBC, and transferrin levels were recorded in the LA-treated group compared to the control group. On the other hand, the LA + CUR and LA + CIN groups revealed an observable decline in serum iron, ferritin, and transferrin saturation percentage besides significant elevations in TIBC, UIBC, and transferrin levels compared to the LA-treated group (Figure 3). It was concluded that LA caused a disturbance in iron parameters which returned toward normal in the CIN- and CUR-treated groups.

### 3.3 Serum immunoglobulins

**FIGURE 4:**
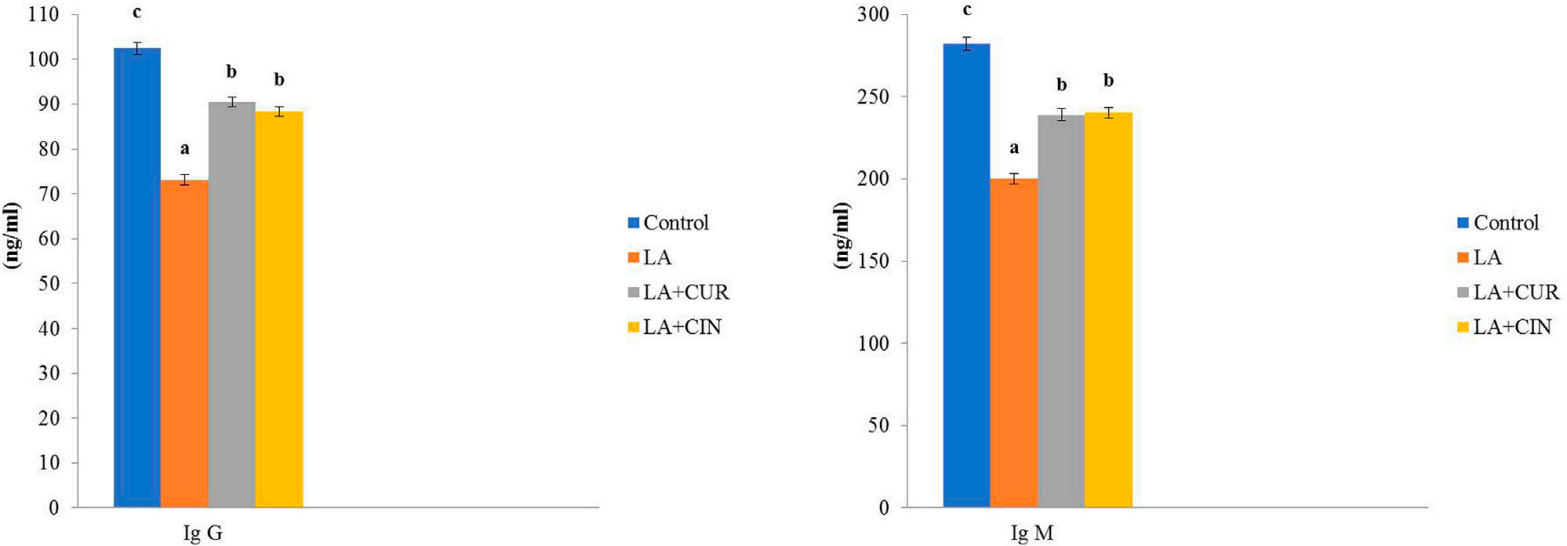
Changes of immunoglobulins in different treatment groups compared to the control (mean ± SE). Different superscript letters in each figure indicate significant differences at *p* < .05.

In comparison to the control group, LA-treated rats exhibited a significant decline in both IgG and IgM levels. Meanwhile, their levels revealed significant increases in the LA + CUR and LA + CIN groups compared to the LA-treated group (Figure 4).

### 3.4 Splenic oxidative/antioxidant parameters

**FIGURE 5:**
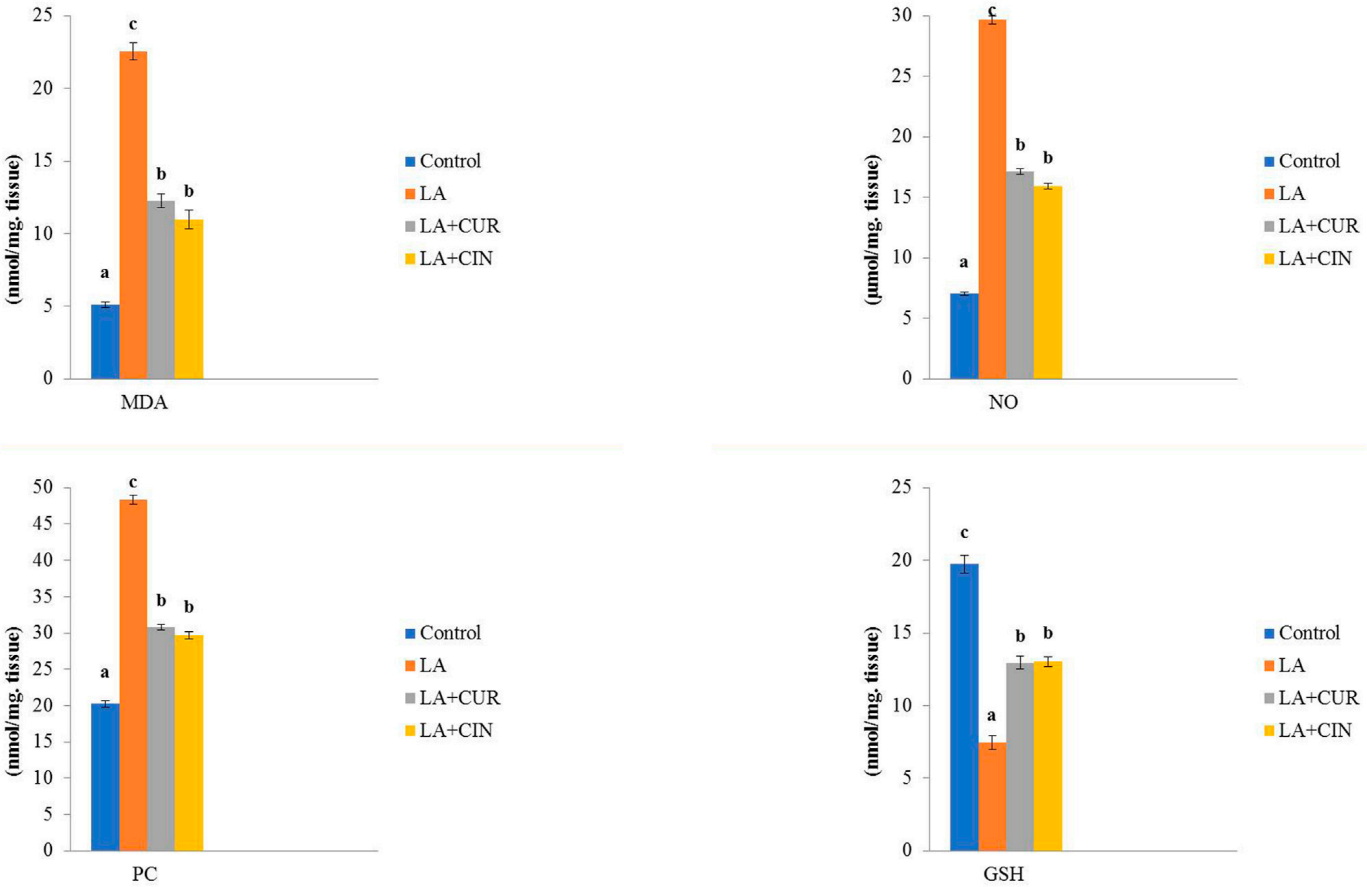
Changes of oxidative stress/antioxidant parameters in different treatment groups compared to the control (mean ± SE). Different superscript letters in each figure indicate significant differences at *p* < .05.

Rats exposed to LA showed significant increases in MDA, NO, and PC, along with a significant reduction in GSH activity compared to the control group. Meanwhile, the LA + CUR and LA + CIN groups exhibited significant declines in MDA, NO, and PC levels as well as a significant elevation in GSH activity compared to the LA-treated group (Figure 5). LA induces oxidative stress and depletion of antioxidants within splenic tissue, but CIN and CUR ameliorated these effects.

### 3.5 Lead content in splenic tissues

**FIGURE 6:**
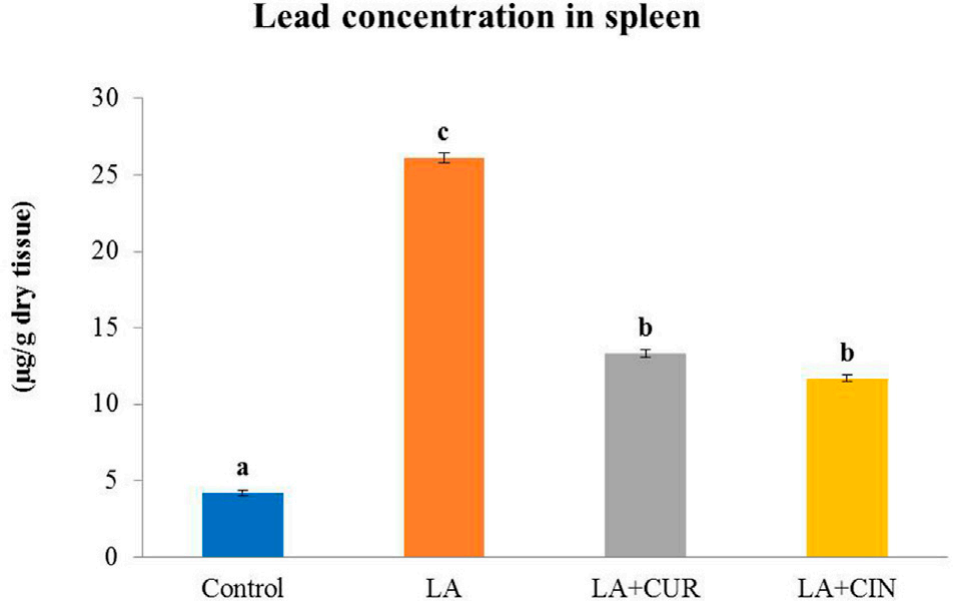
Lead concentration in the splenic tissue of different treatment groups compared with the control (mean ± SE). Different superscripts **(A–C)** in each figure indicate significant differences at *p* < .05.

Splenic tissues from the LA-treated group exhibited a significantly high lead content compared to those from the control group. Meanwhile, groups supplemented with CUR and CIN revealed significantly lower splenic content of lead (Figure 6) compared to the LA-treated group.

### 3.6 Splenic expression of CYP-2E1

**FIGURE 7:**
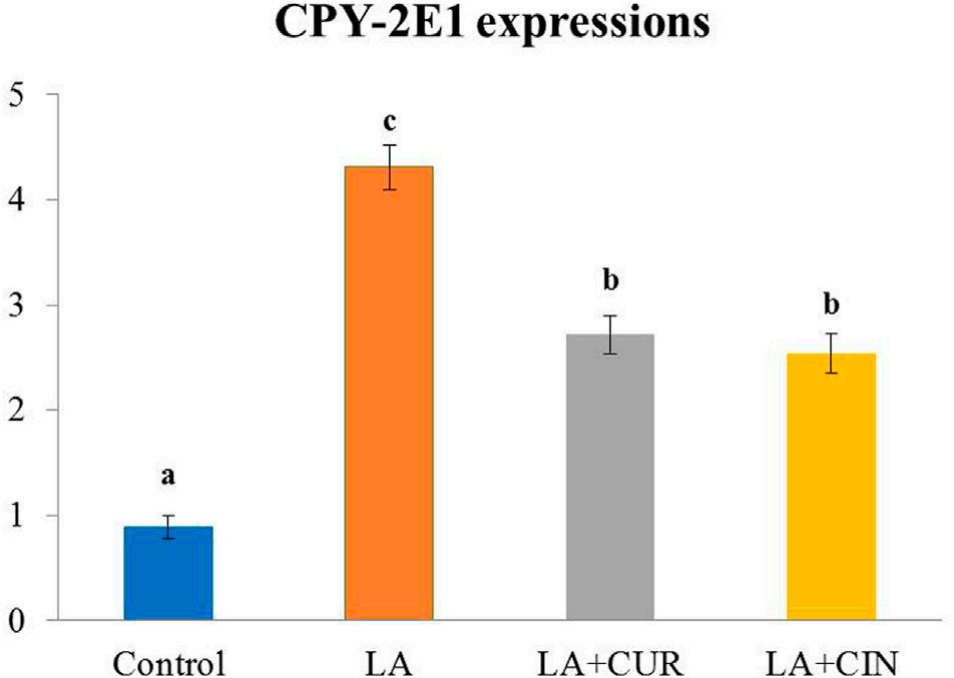
Splenic expression of CYP 2E1 in different treatment groups compared with *ß*-actin (mean ± SE). Different superscripts **(A–C)** in each figure indicate significant differences at *p* < .05.

The splenic expression of CYP-2E1 was upregulated in the LA-treated group compared with the control group. On the contrary, the CUR- and CIN-treated groups revealed significant decreases in the expression of CYP-2E1 compared with the LA-treated group (Figure 7).

### 3.7 Histological findings

**FIGURE 8:**
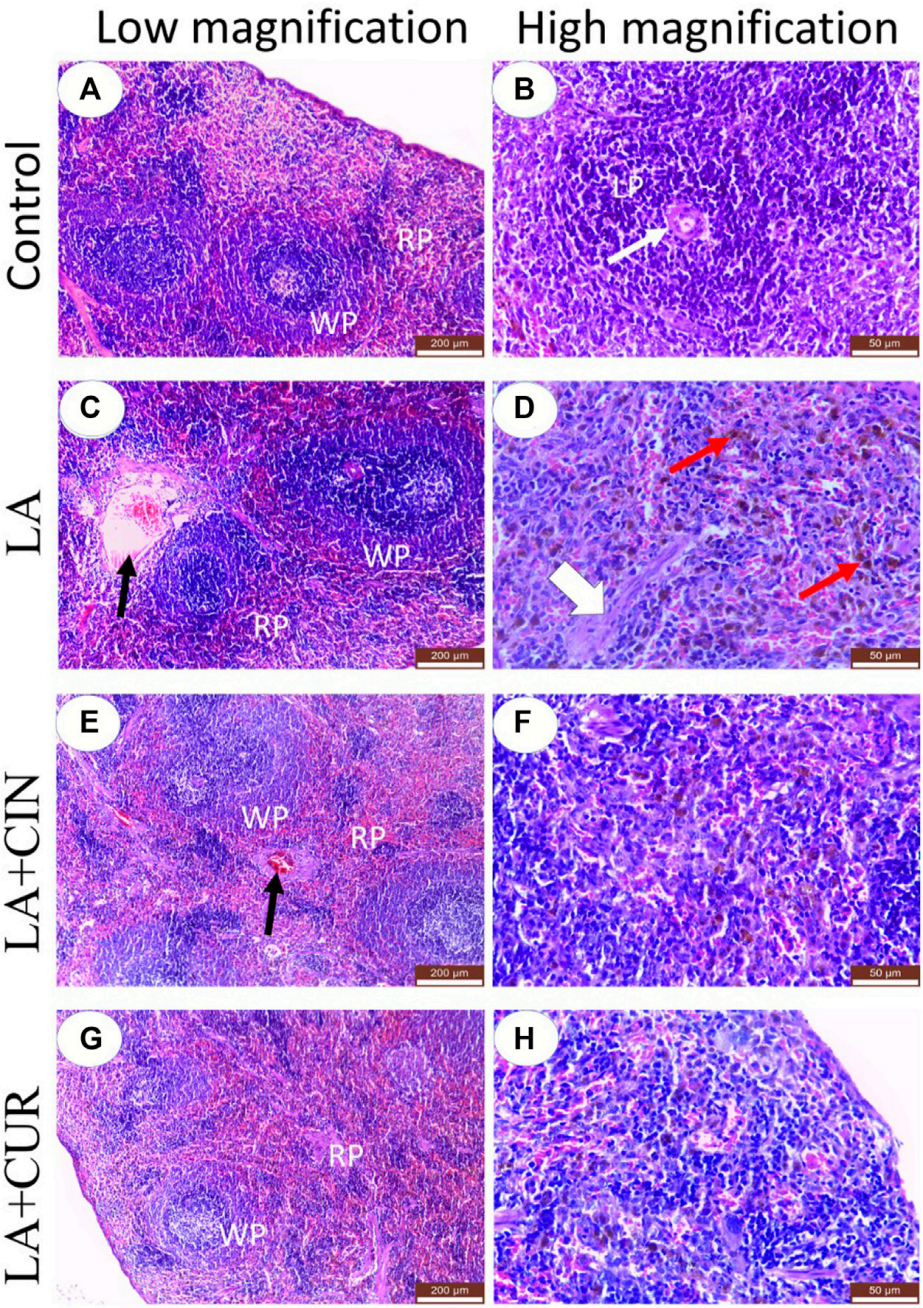
H&E-stained sections of the spleen from adult Albino rats, **(A)** control, **(C)** LA, **(E)** LA + CIN, and **(G)** LA + CUR groups. **(B)**, **(D)**, **(F),** and **(H)** were higher magnification of control, LA, LA + CIN, and **(G)** LA + CUR groups, respectively. Red pulp (RP), white pulp (WP), lymphoid follicle (LP), central arteriole (thin white arrows), blood vessels (black arrows), septa (broad white arrow), and hemosiderin pigments (red arrows). H&E stain.

Splenic tissue sections obtained from the control group revealed normal histo-architecture of red and white pulps (Figure 8A). The splenic red pulp was formed of branching splenic cords and vascular sinusoids. Meanwhile, the white pulp was formed of lymphoid follicles containing closely packed lymphocytes with an eccentrically located arteriole (Figure 8B).

In the LA group, marked histological alterations were detected within the splenic tissue. Significant enlargement of splenic white pulp due to lymphatic follicle proliferation was observed in addition to dilatation of central arterioles (Figure 8C). The splenic red pulp revealed a loss of architecture with significant congestion of blood sinusoids and dilated blood vessels (Figure 8C). Significant cytoplasmic depositions of hemosiderin pigments in many cells of red pulp, in addition to thickened splenic septa, were noticed (Figure 8D).

Both the LA + CIN- and LA + CUR-treated groups revealed a significant improvement of splenic tissue architectures with partially normal white and red pulps compared to the LA group (Figures 8E,G), where prominent red pulps were detected, indicating a reduction of the hyperplastic lymphoid reaction (Figures 8E,G). In comparison to the LA group, the splenic blood vessels showed significantly slight congestion in the CIN + LA group (Figure 8E), and no congestion in the LA + CUR group (Figure 8G) along with significantly slight dilatation of sinusoids with scanty hemosiderin pigment deposition were noticed (Figures 8F,H). A summary of the scoring of histological alteration in splenic tissues among different groups is recorded in Table 2.

**TABLE 2:** Ordinal scoring of splenic histological changes among different groups.

Parameters	Experimental groups			
	Control	LA	LA + CIN	LA + CUR
Enlargement white pulp	.0 ± 0.0d	3.50 ± .07a	.85 ± .10b	.0 ± 0.0c
Dilatation of central arteriole	.0 ± 0.0b	.90 ± .08a	0.0 ± 0.0b	.0 ± 0.0b
Congestion of blood sinusoids	.0 ± 0.0c	3.75 ± .13a	1.40 ± .08b	1.20 ± .06b
Congestion blood vessels	.0 ± 0.0c	1.20 ± .06a	.45 ± .11b	.0 ± .00c
Dilatation of blood vessels	.0 ± 0.0b	1.60 ± .11a	.0 ± 0.0b	.0 ± 0.0b
Thickened splenic septa	.0 ± 0.0b	2.48 ± .07a	.0 ± 0.0b	.0 ± 0.0b
Hemosiderin pigments	.45 ± .12c	2.70 ± .05a	1.60 ± .05b	1.10 ± .11b

LA, lead acetate; CIN, cinnamon; CUR, curcumin.

All values expressed as the mean ± SE. Superscript letters within the same rows were significant (*p* ≤ .05).

### 3.8 Immunohistochemical findings

**FIGURE 9:**
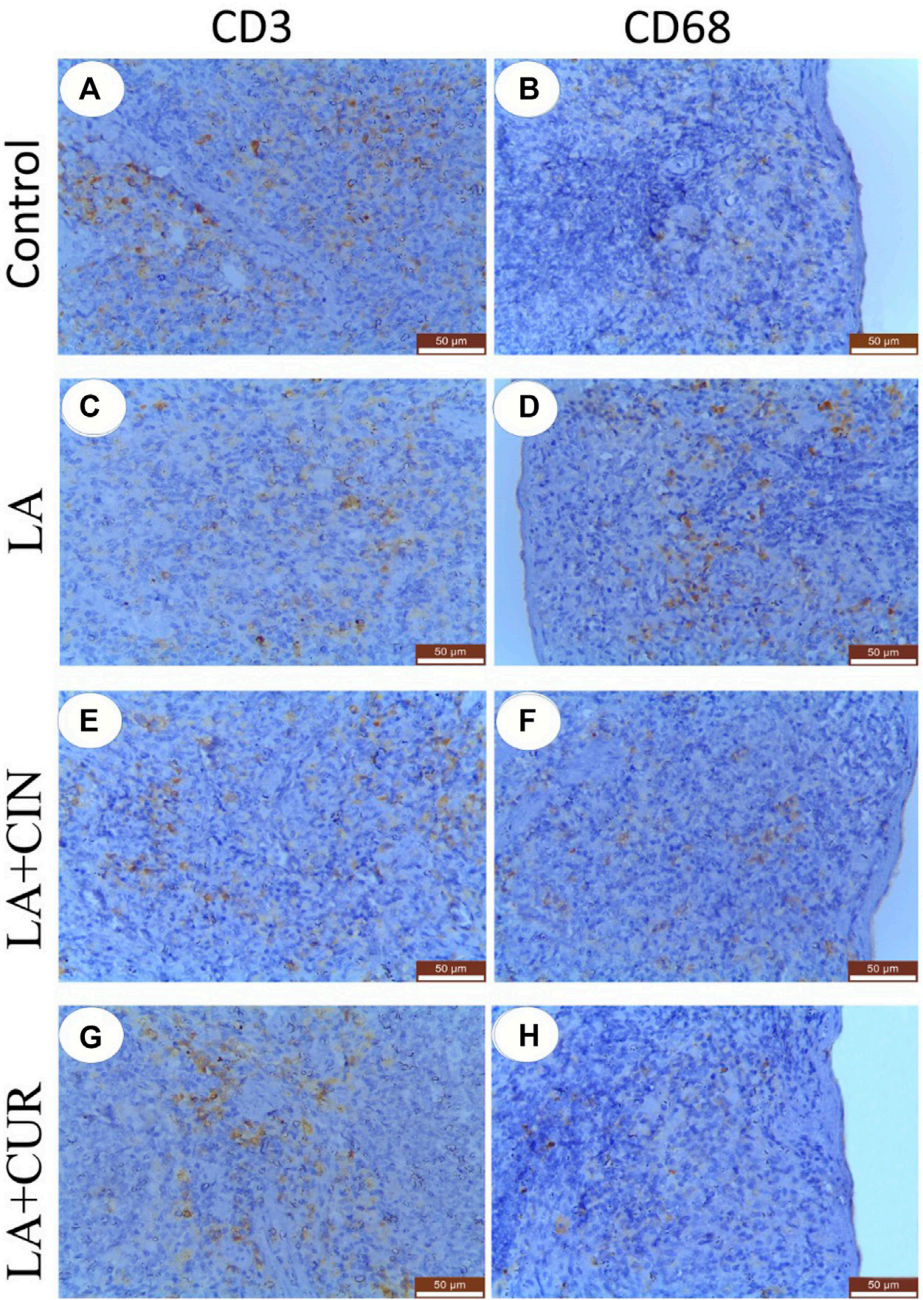
Immunohistochemically stained sections of the spleen with CD3 **(A,C,E, and G)** and CD68 **(B,D,F, and, H)** from different groups. **(A)** control group showed strong CD3 immunostaining, **(C)** LA group showed weak CD3 immunostaining, **e** and **g**; LA + CIN, and LA + CUR groups showed moderate CD3 immunostaining, **(B)** and **(H)**; control and LA + CUR groups showed weak CD68, **(D)**; LA group showed strong CD68 immunostaining, and **(F)**; LA + CIN group showed moderate CD68 immunostaining.

**FIGURE 10:**
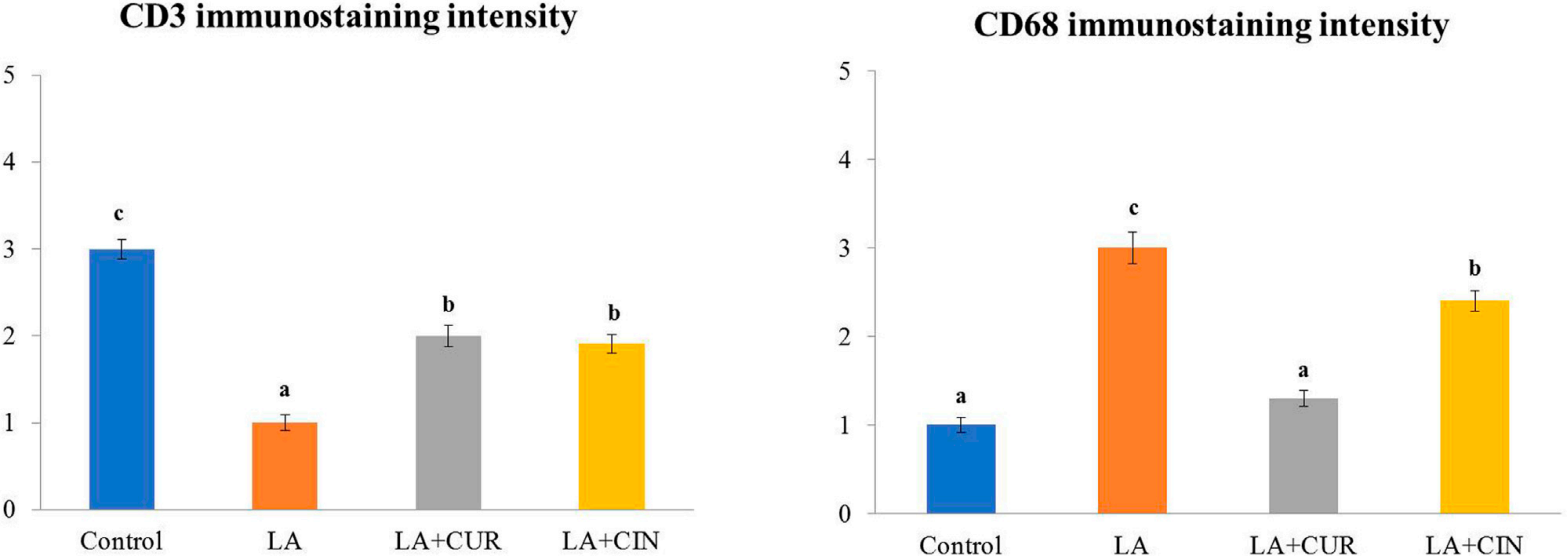
Immunohistochemical intensities of CD3 and CD68 in the spleen of all experimental groups.

The positive immunohistochemical reactions for CD3 and CD68 appeared as brown cytoplasmic staining within splenic tissue cells (Figure 9). A summary of the scoring of splenic CD3 and CD68 expressions among different groups is recorded (Figure 10).

#### 3.8.1 CD3

Splenic sections from the control group revealed significantly strong immunostaining for CD3 (Figure 9A) compared to other groups. Otherwise, a significantly weak CD3 immunostaining was noticed in splenic tissues of the LA group (Figure 9C) compared to control rats. However, significantly moderate immunostaining of CD3 was apparently demonstrated in spleens of both the LA + CIN and LA + CUR groups compared to the LA group (Figures 9E,G).

#### 3.8.2 CD68

A significantly weak CD68 immunostaining in the splenic tissues of both control and LA + CUR groups was noticed (Figures 9B,H). Meanwhile, significantly strong CD68 immunostaining was detected in the spleen of the LA group (Figure 9D) compared to other groups, whereas moderate CD68 immunostaining was seen in the LA + CIN group (Figure 9F).

## 4 Discussion

Lead is one of the most widespread heavy metals in the environment; hence, lead poisoning is a common case. Nowadays, attention has been paid to using herbal medicines instead of synthetic ones due to their low cost, availability, and fewer side effects. Therefore, the current study aims to investigate the potential efficacy of CIN and CUR, as herbal medicine, on hemato-biochemical, immunological, and oxidative stress markers, along with splenic histology and immunohistochemistry after exposure to LA.

Generally, heavy metal exposure can cause damage to the hematopoietic system, resulting in a disturbance in the hematological picture of both animals and humans (WHO, 1995). In the current study and after exposure to LA, rats exhibited significant reductions in RBC count, Hb concentration, PCV percentage, MCH, and MCHC, besides a significant increase in MCV compared to the control group, revealing macrocytic hypochromic anemia. Similar results were reported by Ekanem et al. (2015) and Ismail (2017). Additionally, Hossain et al. (2015) reported that LA induces hemolytic anemia.

LA-induced anemia could be attributed to binding lead to proteins in RBCs (García-Niño and Pedraza-Chaverri, 2014) as well as to shortening of RBCs’ life-span *via* depletion of the antioxidant system within the RBCs (Patra and Swarup, 2000; Lavicoli et al., 2003), inhibition of some Hb synthesizing enzymes (Dongre et al., 2011), and induction of RBC hemolysis *via* Hb oxidation (Flora et al., 2012).

All the previous erythrogram parameters showed an improvement in the LA + CUR and LA + CIN groups compared to the LA-treated group. The improvement of the erythrogram by CUR was in harmony with Ahmad et al. (2015) and El-Maddawy and El-Sayed (2018), where CUR could enhance erythropoiesis and stabilize cell membranes, preventing cellular damage (Banji et al., 2011). CUR may also protect Hb from oxidation (Unnikrishnan and Rao, 1992), and it could increase the intracellular glutathione, which interacts with ROS and free radicals, converting them into an oxidized form Al-Attar, (2022), besides the ability of CUR to improve the absorption of iron from the gut or by facilitating the reduction of oxidized iron to its reduced form (Wardlaw, 1990).

Leukogram results revealed significant elevations in WBC, neutrophil, and monocyte counts and a significant reduction in lymphocyte count in the LA-treated group compared to the control group. This was in agreement with Mugahi et al. (2003). Also, Oyem et al. (2021) founded that LA induces leukocytosis and neutrophilia. Leukocytosis might be considered a response of the phagocytic cells of the blood (neutrophils and monocytes), which engulf the denatured Hb and damaged RBCs (Ekanem et al., 2015) and/or might be due to inflammation induced by LA (Alwaleedi, 2015). Concerning lymphopenia, it was in accordance with Ismail (2017), that may be attributed to immunosuppression induced by LA (Ercal et al., 2001), splenotoxicity (Rocha et al., 2016), or damage of mitochondria and lysosomes in circulating lymphocytes (Zarei et al., 2017).

In contrast, LA + CUR and LA + CIN groups showed significant decreases in WBC, neutrophil, and monocyte counts with a significant increase in lymphocyte count compared to the LA-treated group. This might be attributed to the ability of CUR to modulate the activity of neutrophils and restoration of WBCs (Banji et al., 2011). Furthermore, the increase in lymphocytes might be due to activation of the immune system, stimulating lymphopoiesis (Cetin et al., 2010).

Regarding the ameliorative effect of CIN on LA-induced blood picture disturbance, Ghonim et al. (2017) recorded a similar role of CIN on both erythrogram and leukogram, owing to the antioxidant properties of CIN polyphenols and flavonoids that act as scavengers for reactive oxygen and nitrogen species, besides its effects on chelation of metal and modulation of enzymes (Azab et al., 2011).

Concerning the serum iron parameters, there were significant elevations in serum iron and ferritin levels as well as transferrin saturation percentage, along with significant decreases in TIBC, UIBC, and transferrin levels in the LA-treated group compared to the control group. Our results partially were in harmony with those of Chen et al. (2019a), who reported decreased levels of transferrin and TIBC in lead workers compared to unexposed ones. Elevated serum iron, ferritin, and transferrin saturation percentage might be due to hemolytic anemia induced by LA, where large quantities of iron are released from destroyed RBCs, consequently increasing ferritin level and transferrin saturation percentage. The high serum iron level, accompanied by high ferritin level and low transferrin level, confirms the inverse relationship between ferritin and transferrin levels (Majoni et al., 2017). The LA + CUR and LA + CIN groups revealed an observable decline in serum iron, ferritin, and transferrin saturation percentage, besides significant elevations in TIBC, UIBC, and transferrin levels compared to the LA-treated group. This might be owed to the antioxidant and chelator effects of both CUR and CIN to free iron (Jiao et al., 2006; Azab et al., 2011), where CUR contains chemical groups such as the *ß*-diketonate group (Bernabe-Pineda et al., 2004).

Regarding oxidative stress, ROS, including singlet oxygen (O^−^), hydroxyl radical (OH^+^), and hydrogen peroxide (H_2_O_2_), are generated naturally in various body cells during normal cellular respiration (Le Bras et al., 2005; Small et al., 2012).

These ROS are cytotoxic molecules even during normal cellular respiration, so they should be naturally neutralized by an endogenous antioxidant defense system, including GSH, MDA, and NO (Le Bras et al., 2005; Avery, 2011; Small et al., 2012). If there is an imbalance between ROS generation and antioxidants, severe oxidative stress-induced cellular damage occurs. The excess ROS causes cell membrane and DNA damage, lipid peroxidation, and protein oxidation, leading to various forms of cellular toxicity (Small et al., 2012; Farouk et al., 2021a; b). In the current study, LA-induced oxidative damage involves first ROS generation and then the destruction of the body’s antioxidant defense system (Barbouti et al., 2020). One of the reasons for increasing levels of MDA in the splenic tissue was the increased ROS traversing the cell membrane and destroying neighboring cells, leading to cellular damage of the spleen (Rocha et al., 2016). Lead also renders the cellular GSH inactive, so it becomes unable to restore the GSH supply. Moreover, lead inactivates δ-aminolevulinic acid dehydratase (ALAD), glutathione reductase (GR), glutathione peroxidase (GP_X_), and glutathione-S-transferase, which depresses the glutathione levels (Carocci et al., 2016).

PC is a carbonyl product formed as a result of protein oxidation, where ROS could attack amino acid residues (Harishekar and Kiran, 2011). Owing to the chemical stability of PC, it is easily detectable, and its increase takes place earlier than MDA from lipid peroxidation (Del Rio et al., 2005; Erdogan et al., 2006). Levine et al. (1990) reported that PC derivative formation is associated with some pathological conditions, including rheumatoid arthritis, atherosclerosis, neurological disorders, cataractogenesis, and ischemia. In the case of LA exposure, protein oxidation is predominant, so PC is increased (Rouach et al., 1997).

Rats exposed to LA showed significant increases in MDA, NO, and PC, along with a significant reduction in GSH activity compared to the control group. These results agreed with those of El-Sherbini et al. (2016), El-Tantawy (2016), Ismail (2017), and Oyem et al. (2021). Lead inhibits the cellular thiol antioxidant capacity (Ercal et al., 1996; Gürer et al., 1998). In addition, lead might cause oxidative stress by inhibiting d-aminolevulinic acid dehydratase (ALAD), leading to auto-oxidation of ALAD, forming H_2_O_2_ (Gürer et al., 1998), resulting in oxidative stress and depletion of the antioxidant system. On the other hand, the LA + CUR and LA + CIN groups exhibited significant declines in MDA, NO, and PC levels as well as a significant elevation in GSH activity compared to the LA-treated group. The protective effect of CUR against LA intoxication was a result of its attenuation to antioxidant depletion (Del Rio et al., 2005), scavenging ROS, and chelating LA (García-Niño and Pedraza-Chaverri, 2014; Hismiogullari et al., 2015), that increases the intracellular glutathione concentration, consequently protects lipid peroxidation, and reduces oxidative tissue stress (Okada et al., 2001) and inflammatory damages (Ali et al., 2018). CUR also enhances detoxifying enzyme activity (Abdou and Hassan, 2014).

Regarding the protective effect of CIN, our result was in harmony with that of Elshopakey and Elazab (2021), who reported that CIN can reduce MDA and NO and elevate GSH levels against oxidative stress induced by diclofenac sodium and oxytetracycline. The protective activity of CIN may be attributed to its polyphenol and flavonoid content that acts as ROS scavengers, metal chelators, and enzyme modulators (Azab et al., 2011). Moreover, the active components of CIN such as cinnamaldehyde, cinnamic acid, and eugenol are the principles of its antioxidant and free radical scavenging properties (Gülçin 2011; Jayaprakasha and Rao 2011). Additionally, the reduction in NO level by CIN might be owed to its anti-inflammatory effect associated with its various active components, including cinnamic aldehyde, cinnamyl aldehyde, and tannins, inhibiting the production of NO (Lin et al., 2008).

Regarding the immunoglobulin levels, the LA-treated group revealed a significant reduction in IgM and IgG levels, which was in accordance with Soliman et al. (2015) and Ismail (2017), that might be owed to the effect of LA-induced oxidative stress on B cell function (Hsiao et al., 2011). Such oxidative stress causes Th cell disturbance and alters the availability of Th1 and Th2 cytokines (Hsiao et al., 2011). Meanwhile, IgG and IgM levels revealed significant increases in the LA + CUR and LA + CIN groups compared to the LA-treated group. Similarly, Ciftci (2011) reported that CUR enhances IgG and IgM levels, referring to its immuno-stimulant impact *via* activation of macrophages, neutrophils, natural killer cells, and dendritic cells (Jagetia and Aggarwal, 2007), besides its stimulatory property on humoral immunity (Kim et al., 2013). On the other hand, CIN revealed an improvement in immunoglobulin levels, especially IgG, which agreed with Elshopakey and Elazab (2021). Also, Niphade et al. (2009) reported that CIN has a humoral immunity stimulatory effect *via* elevation of serum immunoglobulins levels through its cinnamaldehyde, benzaldehyde, cuminaldehyde, and terpenes.

Lead concentration in the splenic tissue of LA-exposed rats was significantly high. However, rats supplemented by CUR and CIN revealed lower splenic lead concentration than those in the LA group, and this might be attributed to the metal-chelating property of CUR and CIN (Azab et al., 2011; Soliman et al., 2015).

CYP-2E1 enzyme is one of the cytochrome P450 involved in oxidative stress and lipid peroxidation (Martikainen et al., 2012). CYP-2E1 can metabolize toxic substrates, producing excess ROS (Danielson, 2002), which degrades the CYP hemeprotein releasing iron, potentiating lipid peroxidation (Caro and Cederbaum, 2004). In the present study, LA-induced overexpression of the splenic CYP-2E1 gene agreed with Chen et al. (2019b). On the other hand, administration of CUR and CIN was able to inhibit the upregulation of CYP-2E1 expression in the spleen of treated rats. Also, Eassawy et al. (2021) reported that CUR reduces the expression of CYP-2E1 in the liver of acetaminophen-intoxicated and gamma-irradiated rats.

The hemato-biochemical, immunological, oxidative stress, and gene expression results were confirmed by the histological examination of splenic tissues. The spleen of the control group revealed normal histo-architecture of both white and red pulps, as mentioned by Alkhedaide, (2016). LA exposure induced marked distorted splenic architecture, which was represented by significant hyperplasia of lymphoid follicles and its diffusion into the red pulp as well as depositions of hemosiderin pigment in many cells, which matched with Ekanem et al. (2015), Aldahmash and El-Nager (2016), and Hasan et al. (2016). Hemosiderin pigment might be a result of the engulfment of RBCs by macrophages, indicating LA-induced hemolytic anemia and chronic congestion. Spleen macrophage plays a critical role in iron metabolism *via* iron recycling from damaged or senescent erythrocytes (Wang et al., 2010). Although these histological changes were detected in all treated groups, it was mild and less prominent in both the LA + CIN and LA + CUR groups, confirming the findings of Elgawish and Abdelrazek (2014), who recorded a protective effect of CIN against LA-induced dysfunction of the testes.

Immunohistochemical findings of the current study revealed that LA induced a significant reduction in CD3 and a significant increase of CD68 expressions in the splenic tissues, confirming the immunosuppressive effect of lead (Hsiao et al., 2011; Rocha et al., 2016) and its stimulatory effect on phagocytic cells (Mugahi et al., 2003), respectively. Otherwise, the LA + CIN and LA + CUR groups revealed an increase of CD3 and a reduction of CD68 expressions in splenic tissues compared to the LA group, indicating an immuno-enhancement effect of both CUR and CIN (Banji et al., 2011; Lee et al., 2013; Elshopakey and Elazab, 2021).

In conclusion, the current study figured out the potential ameliorative effect of CIN and CUR upon LA-induced changes in hemato-biochemical parameters and oxidative stress markers, as well as the histology of the spleen and CD3 and CD68 expressions. These results indicate antioxidant, immunomodulatory, and gene-regulating effects of CUR and CIN against LA-induced splenotoxicty in rat models.

## Data Availability

The original contributions presented in the study are included in the article/Supplementary Material; further inquiries can be directed to the corresponding author.
